# ReachCare Mobile Apps for Patients Experiencing Suicidality in the Emergency Department: Development and Usability Testing Using Mixed Methods

**DOI:** 10.2196/41422

**Published:** 2023-01-27

**Authors:** Celine Larkin, Soussan Djamasbi, Edwin D Boudreaux, Fatima Varzgani, Roscoe Garner, Mariam Siddique, John Pietro, Bengisu Tulu

**Affiliations:** 1 Department of Emergency Medicine University of Massachusetts Chan Medical School Worcester, MA United States; 2 The Business School Worcester Polytechnic Institute Worcester, MA United States

**Keywords:** suicide, emergency department, mobile app, usability, engagement, mobile phone

## Abstract

**Background:**

Many individuals with suicide risk present to acute care settings such as emergency departments (EDs). However, staffing and time constraints mean that many EDs are not well equipped to deliver evidence-based interventions for patients experiencing suicidality. An existing intervention initiated in the ED for patients with suicide risk (Emergency Department Safety Assessment and Follow-up Evaluation [ED-SAFE]) has been found to be effective but faces trenchant barriers for widespread adoption.

**Objective:**

On the basis of the ED-SAFE intervention, we aimed to develop 2 apps for patients with suicide risk: a web app guiding patients through safety planning in the ED (*ED app*) and a smartphone app providing patients components of the ED-SAFE program on their phones after discharge (*patient app*). We then tested the usability of these apps with patients presenting to the ED with suicide risk.

**Methods:**

Using a user-centered design framework, we first developed user personas to explore the needs and characteristics of patients who are at risk for suicide using inputs from clinicians (n=3) and suicidologists (n=4). Next, we validated these personas during interviews with individuals with lived experience of suicidality (n=6) and used them to inform our application designs. We field-tested the apps with ED patients presenting with suicide risk (n=14) in 2 iterative cycles to assess their usability and engagement using a mixed methods approach. We also rated the quality and fidelity of the safety plans created.

**Results:**

We developed 2 interoperable and complementary apps. The first is a web app designed for use on a tablet device during ED admission that guides the patient by creating a safety plan using a chatbot-style interface. The second is a smartphone app for use after discharge and allows the patient to view, edit, and share their completed safety plan; access self-care education, helplines, and behavioral health referrals; and track follow-up appointments with the study clinician. The initial prototype usability testing (n=9) demonstrated satisfactory scores (ED app System Usability Scale [SUS], mean 78.6/100, SD 24.1; User Engagement Scale, mean 3.74/5, SD 0.72; patient app SUS, mean 81.7/100, SD 20.1). After refining the apps based on participant feedback, the second cycle testing (n=5) showed improvement (ED app SUS, mean 90.5/100, SD 9.9; User Engagement Scale, mean 4.07/5, SD 0.36; patient app SUS, mean 97.0/100, SD 1.9). The quality ratings for completed safety plans were satisfactory (Safety Planning Intervention Scoring Algorithm-Brief, mean 27.4, SD 3.4).

**Conclusions:**

By adopting a user-centered approach and creating personas to guide development, we were able to create apps for ED patients with suicide risk and obtain satisfactory usability, engagement, and quality scores. Developing digital health tools based on user-centered design principles that deliver evidence-based intervention components may help overcome trenchant implementation barriers in challenging health care settings.

## Introduction

### Background

Suicide is a leading cause of death in the United States [1], devastating families and communities and contributing to the recent decrease in the average life expectancy in United States [2]. Health systems are struggling with the burden of suicide-related presentations, which now represent 1.5 million emergency department (ED) visits each year [3]. A significant proportion of those who died by suicide have visited an ED in the year before their death [4], and about one-eighth of general ED patients had endorsed suicidality when asked [5,6].

Effective interventions for patients experiencing suicidality exist. One such intervention, the Emergency Department Safety Assessment and Follow-up Evaluation (ED-SAFE) [7], led to a 28% reduction in suicide attempts in the year after an ED visit among patients who had recent suicidal ideation or attempt. The ED-SAFE intervention consisted of a self-administered paper-based safety plan and behavioral health referral resources during the visit, followed by supportive counseling phone calls for the patient and a family member over a 12-month period. These follow-up phone calls focused on reinforcement and refinement of the safety plan, establishment of a plan for outpatient behavioral health treatment engagement, value clarification, development of a life plan for value-based living, and family engagement. Other effective interventions for patients who are experiencing suicidality include clinician-administered safety planning interventions [8,9] and caring contacts [10,11]. However, such interventions have shown limited adoption and reach in busy EDs [12], as ED resources are limited and the primary clinical goal is the evaluation, stabilization, and appropriate disposition of patients [13].

Technology may present a solution to some of the trenchant implementation barriers to evidence-based interventions for suicide risk in the ED. Patient-facing technologies may help to scale up evidence-based care by decreasing the cost, time, staffing, expertise, and overall burden, while preserving the quality and fidelity of the interventions. For example, Boudreaux et al [14] showed that computer-based self-administration of the safety planning intervention with patients who are experiencing suicidality was feasible, generated high-fidelity safety plans, and reduced suicide intensity in the short term.

### Objectives

Given the effectiveness of the original ED-SAFE intervention and feasibility of the technology-based self-administration of its key components, a technology-facilitated delivery of this intervention may help address the needs of ED patients experiencing suicidality. To test this hypothesis, we sought to develop and test a technology-facilitated version of the ED-SAFE intervention, called *ReachCare*, by applying user-centered design (UCD) principles as recommended in the literature and iterative development methodology [15]. Here, we report the initial development and testing of 2 patient-facing apps of ReachCare. The first app is designed to facilitate self-administered safety planning in the ED. The second app was designed to give patients and their families access to the safety plan, behavioral health referrals, and other behavioral health resources after hospital discharge on their own devices. We hypothesized that following UCD principles and iterative development methodologies would allow for the successful development of technology-based solutions that can deliver evidence-based interventions for patients experiencing suicidality in the emergency setting.

## Methods

### Overview

User experience has become a major factor in the development of successful user-centered technological solutions and interventions [16-18]. The UCD framework, which we used to develop and test our technology-based intervention, relies on both generative and evaluative user experience research practices. The generative phase aims to elucidate the needs and goals of individuals for whom a product or service is designed. The evaluative phase focuses on assessing the effectiveness of the designed products and services in meeting user needs and goals. Following this framework, we used an iterative process to design and test 2 applications for ED patients presenting with suicide risk based on the ED-SAFE intervention.

We first developed an initial set of personas to understand patient needs and challenges from a clinician’s point of view and used this information to develop low-fidelity prototypes for the 2 ReachCare applications (iteration 1). Next, we gathered additional information about the needs, challenges, and goals from patients’ point of view to refine our personas and prototypes (iteration 2). We refined our prototypes based on the information gained from user tests in iterations 1 and 2. This resulted in 2 minimum viable products for patients with suicide risk: a web application (the ED app) for use in the ED on a tablet device (or other mobile device with a large screen) and a smartphone app (the patient app) for use after discharge from the hospital (iteration 3). We then tested the design efficacy and usability of these apps in a field user study using 2 iterative cycles.

### Ethical Considerations

This study was approved by the Institutional Review Board of the University of Massachusetts Chan Medical School (H00020238). We received a waiver of written consent for the initial persona development interviews and design feedback sessions. For iteration 3, which involved usability testing with current patients in the ED, we obtained written informed consent from all participants. A research assistant approached potential participants, explained the study, and shared an information leaflet. If the patient was interested and eligible, the research assistant explained the risks and benefits of participation, what the study entailed, procedures to protect confidentiality and privacy, and the patient’s right to withdraw at any time. The patients then completed a consent mini-quiz to demonstrate that they fully understood the information before signing the informed consent form. The participants were given a US $30 gift card as a token of appreciation.

### Iteration 1: Developing Personas

A major objective of persona development is to inform and prioritize design decisions. To achieve this goal, a comprehensive persona development process typically starts by collecting information from a group of experts with firsthand knowledge of the target users. This information is then organized into a set of clusters via an iterative *create and adjust* process until a small set of clusters representing major target user groups is developed. One way to guide the clustering process is by developing a set of user attributes that can help group the information obtained from expert interviews into major themes. Such user attributes are typically developed in collaboration with the expert participants to ensure that they are relevant and important in categorizing target users for the project at hand [17,19].

**Figure 1:**
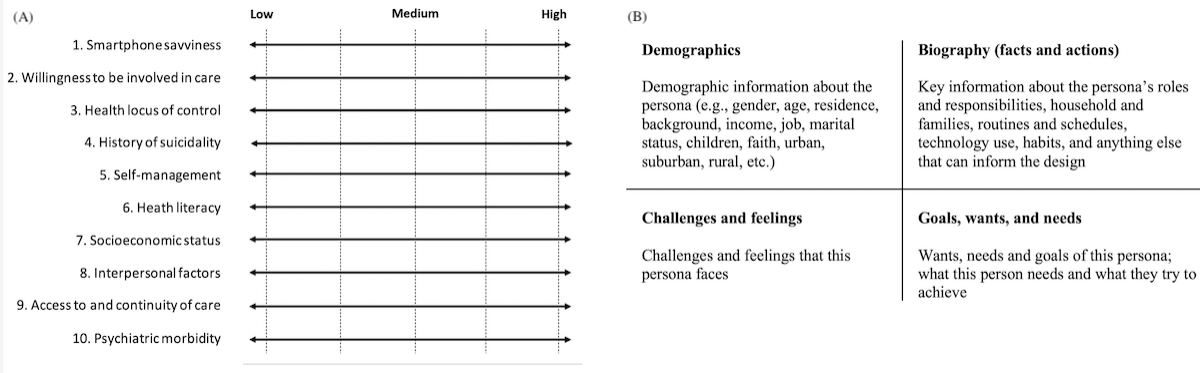
Persona development material: (A) attribute spectrums and (B) worksheet.

To develop the initial set of personas for our project, we gathered information from a group of subject matter experts (4 suicidologists and 3 clinicians) familiar with the needs of users that the ReachCare program intends to serve. This process started by identifying a set of 10 user attributes that were intended to serve as guides for clustering the information obtained from interviews into themes. Next, we shared these attributes (Figure 1A) and their definitions with our subject matter experts via email. The expert participants were then asked to review the provided information, make suggestions on removing or revising the items or their definitions, and suggest any additional user attributes that they may find important to consider for the specified population in our project. All subject matter experts confirmed the relevance and importance of the attributes. None of the experts suggested any revisions or suggested any additional attributes.

Finally, we conducted individual interviews via video conference with the same subject matter expert participants. During the 1-hour interviews, we first asked the participants to complete 2 worksheets (Figure 1B), each worksheet representing a specific group of ED patients with suicide risk whom they had encountered in their practice. The worksheet required respondents to provide information about demographics, daily routines, challenges, feelings, goals, wants, and needs of a persona. After completing 2 worksheets, we presented the participants with the list of attributes that they had reviewed and verified before the interview. We asked them to identify where their developed persona would fall on a low to high spectrum for each of the 10 attributes (Figure 1A).

**Figure 2:**
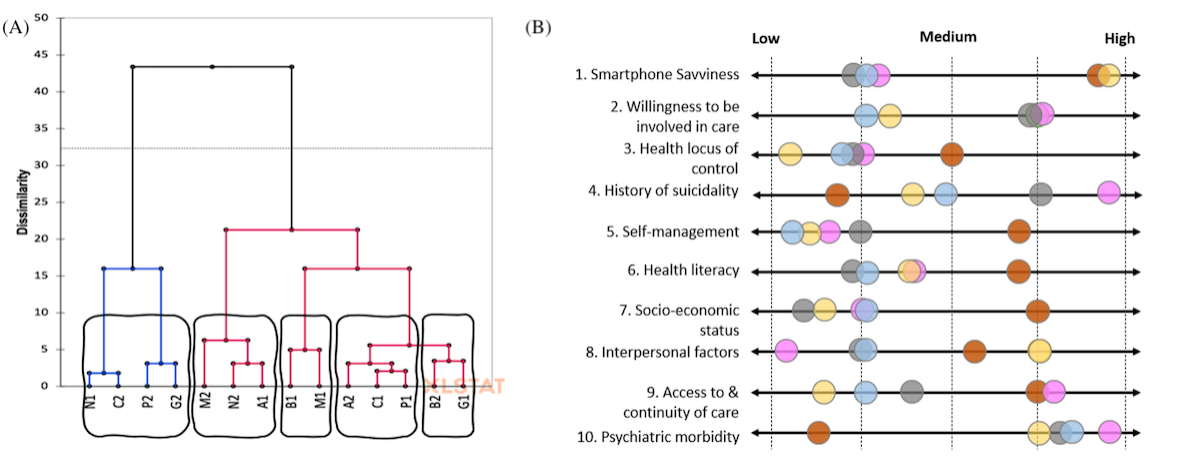
A snapshot of the categorization process: (A) cluster analysis resulting in 5 clusters for the 14 participants (N2, C2, P2, etc), and (B) spectrum map for the 5 identified clusters.

The data collected from the expert interviews resulted in 14 sets of worksheets and their respective ratings along 10 attributes. We used the attribute ratings to organize the completed worksheets into a small set of major user groups. To achieve this objective, we first conducted hierarchical cluster analysis. On the basis of the degree to which the worksheets’ attributes were similar, this analysis suggested that the 14 worksheets could be categorized into 5 clusters (Figure 2A). We then synthesized the captured information in each cluster to identify emerging themes and visualized the synthesized attribute spectrum for each cluster via a spectrum map (Figure 2B).

The manual inspection of the emerging themes from the worksheets in each cluster and their respective attribute spectrum map revealed that 2 of the suggested clusters could be consolidated into 1 to form an overarching user group. Hence, our iterative categorization process resulted in 4 major clusters, each representing a distinct user group. Textbox 1 provides brief descriptions of the 4 distinct personas identified.

This categorization process allowed us to identify user needs that were unique to each persona and those that were shared by all user groups. We used this information to prioritize our design efforts. In this project, we focused on designing for common user needs rather than implementing a solution for a specific user group (ie, we delegated the design of personalized features based on distinct persona needs to our subsequent projects).

The overarching user needs that had to be addressed in our app design included challenges with cognitive processing, emotional dysregulation, lack of belonging, and disempowerment. Hence, our apps had to be designed to elicit minimal cognitive effort and facilitate a calm experience, fostering a caring, supportive, and welcoming relationship with the user. We addressed these user needs with an experience design in our project using the design objectives described in Textbox 2.

**Figure 3:**
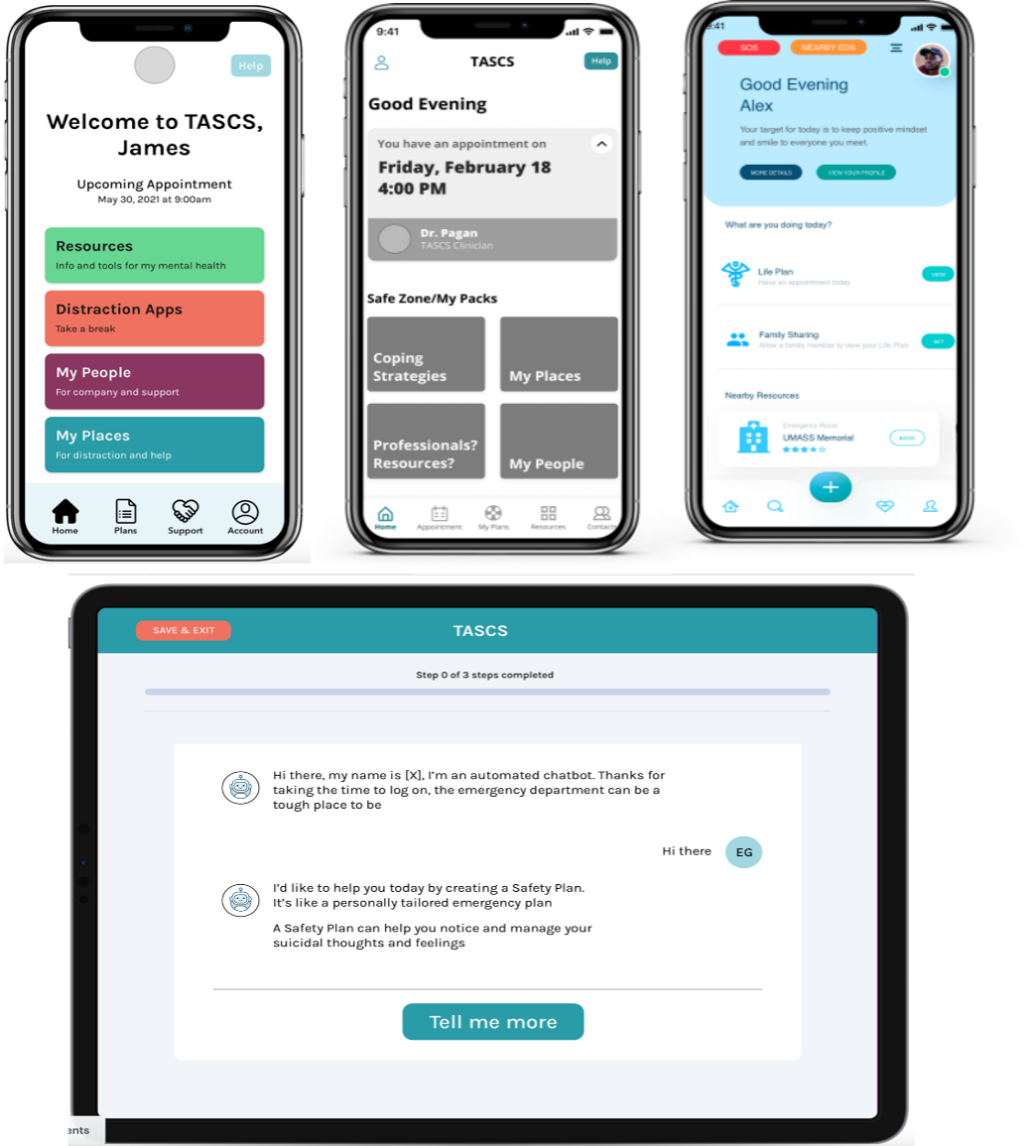
Design mock-ups for the patient and ED apps for iteration 2. ED: emergency department; TASCS: Technology-Assisted Systems Change for Suicide prevention.

We used the above user experience design objectives to create a set of design mock-ups (Figure 3) and 3 introductory videos for the ED app. These materials were then used in iteration 2 to engage a set of target users in an open conversation about their reactions to, and preferences for, our design ideas that address common user needs.

**A brief description for each of the 4 developed personas.**

- Traumatized, lonely, and rejected
  mostly middle-aged adults diagnosed with multiple mental health disorders and trauma. They face challenges living independently due to social and financial issues and value efforts by others to prevent them from harming themselves. They feel rejected by society, believing that nobody cares about them. They experience lower socioeconomic status, often unable to maintain a stable lifestyle.
- Overwhelmed and challenged
  mostly young adults who are overwhelmed by academic, career, and relationship expectations and challenges. They have access to medical and care resources but do not make use of them.
- Rebellious
  includes mostly young people (children and young adolescents) who become rebellious because of lack of control over their lives. Experiencing feelings of powerlessness, their suicidal behavior may have an interpersonal function.
- Substance misuse
  mostly middle-aged adults who have unstable lives due to excessive substance use. They have a limited social life and are reluctant to engage in medical care as they believe health care providers are suspicious of them for coming to the emergency department for secondary gain.

**Design objectives.**

- Reduce cognitive effort
  Because most patients experiencing suicidality would be distressed when they encountered our web-based ReachCare app in the emergency department (ED), we decided to use a conversational user interface that mimics a chatbot. The chatbot approach enabled us to provide information to patients in small digestible pieces. To address this design objective, we also decided to use a minimalistic interface design for both apps. Simple uncluttered screen designs with flat hierarchies and large clearly labeled buttons make the apps easy to learn (intuitive and predictable) and easy to use [20]. We also decided to develop the textual content of both apps at a relatively low reading level to minimize reading effort [21].
- Facilitate a calm experience
  On the basis of previous research, we chose color palettes that could cue a calm and relaxing visual environment [22]. The simplistic uncluttered screen design along with these color pallets could help engage user attention without overstimulation.
- Provide a caring and supportive companionship
  We decided to create an app name that reflected care and support and an app logo that served as a visual cue for signaling care and support. We also decided that the communicative language used in the apps should reflect empathy, caring, and social presence [23]. Supportive, calm, and caring technology design is particularly important for distressed patients who might be viewing the web-based app alone at a time of crisis in the ED.
- Foster a welcoming relationship
  We decided to greet the patients with a video that established a relationship and explained the purpose of the web-based app, as well as the process patients would follow during their ED visit. To address the varying needs of our 4 personas, we decided to record 3 videos, each using a different presenter delivering the same content. One live-action video displayed a female clinician speaking from a clinician’s perspective; another live-action video displayed a male presenter speaking from the perspective of a community member; and one computer-animated video displayed an avatar of ambiguous gender. Delivering the same content through 3 different presenters allowed us to examine which presenter could foster the most comfortable experience.

### Iteration 2: Refining Personas and Testing a Set of Initial Design Mock-ups

In this iteration, we had 2 major goals. First, we wanted to verify and refine our developed personas. Our initial personas were created based on the assumptions of proxy stakeholders (ie, clinicians and suicidologists). To deepen our understanding of patient needs and challenges, personas needed to be refined by eliciting the perspectives of individuals with direct lived experience of suicidality [17,19]. To do so, we prepared interview questions related to persona verification that solicited information regarding patients’ challenges and needs. The second goal in this iteration was to solicit user feedback (eg, preferences and disapprovals) on how our design ideas could address patients’ practical and emotional needs. Hence, we also prepared a set of slides that displayed app screens and visualized the flow and content of our design mock-ups (Figure 3), as well as a set of interview questions to engage participants in a co-design conversation. Our interview questions focused on reactions (likes and dislikes) to the design mock-ups, as well as gathering suggestions for improving the app designs and content to make them more responsive to the needs of the patients with suicide risk in the ED and other settings.

We conducted 90-minute video-conference interviews with individuals (n=6; 3/6, 50% male; age range, 18-73 years; 5/6, 83% White individuals) with lived experience of suicidality, recruited through the existing advisory board and referrals. Informed verbal consent was obtained from all the participants. The first part of the interview was used to gather information to verify and refine our developed personas, and the second half was used to solicit feedback on our design ideas.

The analysis of the interviews verified our initial personas and refined them by revealing how participants felt about ED visits. The participants emphasized that presenting for behavioral health was lonely, frightening, tedious, stigmatizing, and dehumanizing. Conversations facilitated through co-design questions confirmed our design objectives; that is, our applications should be clear, warm, and caring if they were to properly serve those who present with suicidality. The participants thought that the chatbot-style approach to safety planning provided through the ED app would be acceptable. User feedback for the 3 introductory videos revealed that providing choices for content delivery was particularly important for this population. When we asked the participants which introductory video they preferred to see in the ED app, the feedback was mixed. Each video was preferred by at least 1 participant. Some participants showed strong preference for the female presenter, some for the male presenter, and some for the animated character over human presenters. Some participants thought that the animated video was childish and unsuitable for a suicidal crisis. In the animated video, some participants voiced their reservations with the background music because it affected their ability to focus on what the character was saying. Participants with a history of exposure to violence were opposed to the video with the male presenter. Because there was no consensus about which video was best and because participants described a feeling of disempowerment experienced in the ED, a recurring suggestion was to offer a choice of the 3 videos so that the user could follow their own preference.

Participants liked the idea of completing the safety planning through the ED app because it allowed them to think ahead about what could help them in a future crisis. The idea of having the chatbot provide suggestions for some questions was also supported by the participants. According to these participants, the prepopulated suggestions helped them come up with new ideas for content when they were unable to generate content on their own.

For the patient app, the participants showed a strong preference toward the tile-based user interface design of the home page (Figure 3). According to them, it drew more attention than a list-based interface design and made it easier to locate different features owing to bigger and more prominent text and buttons. They also liked the placement of the *help* button for the emergency crisis helpline, as it made the option easily accessible. Other features endorsed by the participants were the sections titled *My People* and *My Plans*, as they provided easy access to the information necessary at the time of crisis. For the distractions feature, a common recommendation was to provide a prepopulated list of activities, such as soothing videos, games, music, or stories, rather than links to certain apps.

### Iteration 3: Developing and Testing High-Fidelity App Prototypes

#### Overview

User feedback on prototypes obtained from user interviews in iteration 2 was used to develop the first minimum viable products for our ReachCare apps. The design efficacy of these high-fidelity prototypes was then tested in another user study. We begin this section by explaining the content and workflow of these high-fidelity prototypes. We then explained how these prototypes were tested in a group of actual patients in the ED.

#### The ED App Content and Workflow

The ED app was developed as a web app for use on any mobile device (eg, a tablet or laptop) during the visit. Before the patients can use the ED app, a staff member must log into the app and onboard the patient by adding their name, email address, and date of birth and then hand the device to the patient. The ED app invited the patient to choose one of three 2-minute introductory videos presented by a live-action female clinician, a live-action male community member, or an animated avatar. The videos briefly explained the concept of a safety plan, how it can be useful to de-escalate suicidal thoughts, and how it can prevent individuals from acting on these thoughts. It also explained how the chatbot in the app would help the patient create a safety plan and how the patient can access their safety plan after discharge on their smartphone via the patient app.

**Figure 4:**
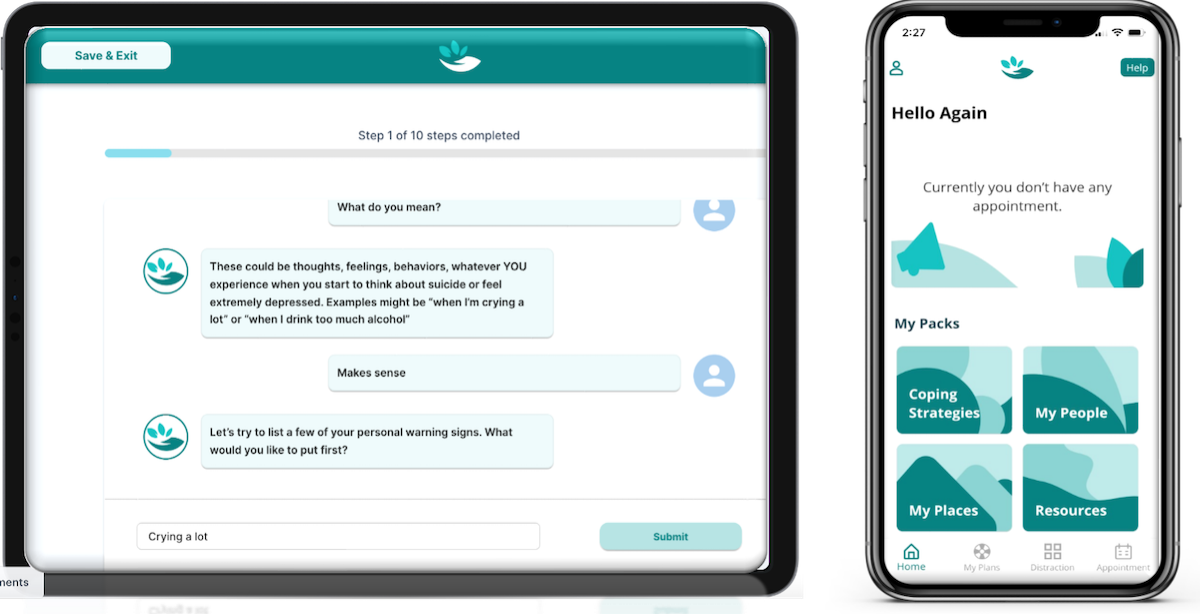
ED and patient apps used in iteration 3. ED: emergency department.

After watching their selected video, the patients were invited to start the safety planning process guided by the chatbot. Our chatbot design was not based on artificial intelligence, was minimally adaptive, and used the 6-step process of the Safety Planning Intervention. The 6 steps were warning signs, coping strategies, people and places for distraction, people for help, professionals for help, and environmental safety. The chatbot communication with patients was visualized via chat bubbles with no more than 2 simple text sentences at a time. Patients hit confirmatory chat bubbles (such as “Got it” and “Ok”) to prompt more instructions to appear (Figure 4). Textbox 3 illustrates an example.

The patient could scroll up to view the earlier text and instructions as needed. For each step of safety planning, the chatbot provided rationale and instructions and then prompted the patient to type in their first responses for that step. This patient-generated text formed the basis of their personalized safety plan. If a patient struggled to think of sufficient content to complete each step, they could opt to select from a set of fixed options provided by the chatbot. The fixed list of options was developed based on the most prevalent responses to safety plans in our previous research [24]. When the patient had completed all 6 steps, the app gave them the opportunity to review their completed safety plan and edit it as desired. Next, the app provided patients with an outro video (delivered by the same character that the patient chose for the intro video) that explained downloading the smartphone app, the care they would receive after leaving the ED, and the next steps in their current visit. Finally, the patient confirmed their email address to receive an email with a link to download the smartphone app.

**Coping strategies, the chatbot-style content, and turn-taking runs.**

- ReachCare response
  “Nice job! Ok, now on to your second step. Can you think of activities that you can do to manage those thoughts and feelings once they arise?”
- Patient response
  “Like what?” button provided for patient to continue
- ReachCare response
  “These would be things that you can do on your own, without needing to contact other people, to take your mind off your problems for a while. For example, “Watching old episodes of Friends” or “Playing with my dog” would be great answers. Does that make sense?”
- Patient response
  “Got it” button provided for patient to continue
- ReachCare response
  “We’re going to try to include some of these coping activities you can do on your own. Try to make them as realistic and specific as possible! What would you like to put first?”
- Patient response
  An *empty text field provided for patient to fill out*.
- ReachCare response
  “What’s another coping strategy or distraction that helps? Try to be as specific as possible.”
- Patient response
  An *empty text field provided for patient to fill out*.
- ReachCare response
  “Can you think of one more distracting or comforting activity you can do on your own?”
- Patient response
  “Yes” button and “No, can you suggest something?” button provided for patient to choose and continue.
    If clicked “yes,” an *empty text field provided for patient to fill out*.
    If clicked “No, can you suggest something?” patient can choose from one of the response buttons (“Take a walk,” “Listen to music,” or “Read a book or magazine”), which was saved as patient response.

#### The Patient App Content and Workflow

The smartphone app was designed for use after discharge from the ED on the patient’s own device. Upon completion of the safety planning workflow in the ED app, the patient received an email with an app store link, their username and password, and instructions to download and log in to the patient app on their smartphone. The patient app contained several key resources. The home screen in the app (Figure 4) provided easy access to key content from the patient’s personalized safety plan, such as Coping Strategies, My People, and My Places. The home screen also provides access to a variety of resources such as (1) a helpline directory, including the national suicide prevention hotline and helplines for at-risk groups, such as veterans and LGBTQ patients; (2) a referral search engine for local behavioral health providers, with options to filter by zip code and specialty; and (3) a collection of self-care education material about topics such as suicidality, safety plans, life plans, behavioral activation, outpatient treatment engagement, and the ReachCare program itself

The icons at the bottom of the screen provided quick access to the Home screen, My Plans, Distractions, and Appointments sections. Under My Plans, patients could view and edit the safety plan they created during the ED visit. The My Plans button also gave the patients access to their Life Plan and Behavioral Health Action Plan. These 2 plans were completed by the patients as part of their follow-up contact with the ReachCare clinician [25]. Under Distractions, the patients could access several original videos that focused on nature, animals, and puzzles. The Appointments screen allowed patients to view their upcoming appointments with the ReachCare clinician. The Help button, placed on the top right corner of most screens, provided a shortcut for the patient to dial emergency services, namely 911 or the National Suicide Prevention Lifeline. Finally, the patient app had a Share Access feature, where a patient could invite their family member to ReachCare and choose which personalized plans they would like to share. After sharing, the family member would receive an email invitation to the app with links to download the patient app on their device as well as their own username and password to login with restricted privileges as described previously. Family members thereby had access (authorized by the patient) to selected patient plans and appointments, as well as the standard helpline, self-care education, distraction, and behavioral health referral information.

#### Usability Testing

The usability testing for our minimum viable products (ie, the high-fidelity ReachCare app prototypes) was conducted in an active ED setting. The ED in question was located within an urban teaching hospital in Massachusetts, with approximately 100,000 patients per year, and included a psychiatric emergency unit. The ED protocol required that all patients be screened for suicide using the Patient Safety Screener [26], regardless of presenting complaints. Eligible patients (1) were aged 18 years or older, (2) were fluent in English, (3) screened positive for suicide risk during triage on the Patient Safety Screener [26], and (4) were cognitively capable of consenting. Patients were excluded if they were (1) overly agitated, (2) too medically ill, (3) a prisoner, or (4) discharged from the ED before they were approached for this study.

### Procedure

After ascertaining the eligibility of the patients and obtaining written informed consent, the research assistant gathered their baseline characteristics, including demographics, mental health history, smartphone use, and smartphone self-efficacy. Next, the research assistant explained to the participants that they could enter either real or test answers to the questions posed by the app (if the patient chose to give real answers, they were offered a paper copy of their safety plan at the end of the session). The participants were then invited to complete the ED app on a tablet and share any feedback aloud as they proceeded, following a think-aloud process. After patients finished working with the ED app, the research assistant verbally administered the 10-item System Usability Scale (SUS) [27] based on that app, as well as the short form version of the User Engagement Scale (UES) [28]. The participants were then invited to view the patient app on a study smartphone and provide feedback as they navigated through the app on their own. After receiving feedback on the app, the research assistant verbally administered the SUS based on the patient app. Completed safety plans were rated by our research team for quality and completeness afterward using the Brief Safety Plan Scoring Form [29]. Patients received a US $30 gift card after the completion of the research interview.

Using this approach, we tested the usability of ReachCare in 2 different cycles. In the first cycle, after we enrolled a set of patients to test the apps, we analyzed their feedback and made improvements based on their suggestions. In the second cycle, we retested the refined apps with a new set of patients until we reached a milestone of the SUS score >80 for 3 patients. We also invited cycle 2 patients to download the patient app on their smartphones and use it for 1 week after discharge. We called the cycle 2 participants a week later to obtain feedback on their experience of using the patient app. Because the app was only available on Google Play Store (Google LLC) at the time, in cycle 2, we only included patients who owned an Android device.

## Results

### Cycle 1

#### Participant Characteristics in Cycle 1

In total, 22 patients were approached by the research assistant in cycle 1; of them, 5 (23%) patients declined, 4 (18%) were being actively discharged, 3 (14%) patients had impairments that rendered them unable to consent, and 10 (45%) patients were enrolled. One of the enrolled patients reviewed the apps but declined to complete the measures and was therefore excluded from these analyses. A total of 41% (9/22) of patients completed the research interview: 33% (3/9) of patients were women and 67% (6/9) of patients were men. Of them, 78% (7/9) of patients were identified as White, 11% (1/9) of patients as Black or African American, and 11% (1/9) of patients as more than one race. A total of 56% (5/9) of participants were Hispanic or Latino. The mean age of participants was 29.2 (SD12.9) years. In addition, 67% (6/9) of participants reported being diagnosed with a psychiatric or emotional disorder in the past. All (9/9, 100%) participants endorsed having thoughts of harming or killing themselves, and all had attempted to kill or harm themselves at some point in their lives. All but one (8/9, 89%) participant owned a smartphone, and 67% (6/9) of participants had brought their cellphones to the ED. However, none of the participants had access to their cell phones at the point of the research interview: the hospital protocol for patients with substantial suicide risk required that cell phones and other personal belongings be removed to avoid in situ self-harm. Participants reported significant phone usage: 56% (5/9) of participants accepted using their cell phones for more than 3 hours each day. In terms of smartphone self-efficacy, 89% (8/9) of participants agreed or strongly agreed that they could use smartphone technology if there was no one around to tell them what to do; 67% (6/9) of participants agreed or strongly agreed that they could use smartphone technology even if they had never used a similar technology before; and all 9 (100%) participants agreed or strongly agreed that they were confident that they could effectively open and use an app on a smartphone. These characteristics were consistent with the personas we identified in earlier iterations.

#### ED App Usability in Cycle 1

Of the 9 participants, 5 (56%) participants chose to view the clinician intro video; 2 (22%) chose the animated video; and 2 (22%) chose the community member video. This result confirms our design choice to provide all 3 videos in the ED app to satisfy a variety of preferences. There was no association or pattern between participant gender and video choice.

Qualitative feedback on the ED app mostly comprised comments on text content and word choice. Some of the questions or statements in the chatbot were seen as repetitive and sometimes reinforced feelings of loneliness (eg, being asked to identify “people for support” when the patient felt that they did not have anyone for support). Hence, the participants recommended more comforting and validating language in some places. Participants suggested making some answers optional (eg, adding phone numbers and addresses of people and places) because they were difficult to remember and not easily accessible during an ED visit. Some participants were confused by the progress bar because it treated the whole safety plan as one “step,” when in fact the safety plan was the most time-consuming part of the process. Apart from chatbot wording, another common recommendation was to add audio accessibility for people with vision issues or those who were not in a state to read a large amount of text. Participants also suggested adding short descriptions to each video option to clarify the purpose and content of each video. Overall, the ED app received positive comments, and all (9/9, 100%) the participants accepted that they would be satisfied to use it. On the basis of these suggestions, we added brief descriptions alongside the introductory videos so that the patient could make an informed decision while selecting the video to play. We also added closed captions to the videos, as the ED environment can be noisy and distracting. We adapted the chatbot wording to reassure the patients that it was alright if they did not have a support person to list in their safety plan. We also updated the progress bar to show more granular progress.

All the participants were able to initiate the videos and navigated successfully through the safety planning process. The mean score of the SUS in cycle 1 for the ED app was 78.6 (SD 24.1) out of a possible 100, indicating that the usability of the ED app was acceptable [30], and the mean UES score was 3.74 (SD 0.72) out of a possible 5. Analysis of the UES subscales showed that perceived usability (mean 4.22, SD 0.93) and reward factor (mean 4.44, SD 0.73) were very high and aesthetic appeal was good (mean 3.70, SD 1.26), while focused attention was lower (mean 2.59, SD 0.78), which verifies the identified need for designing uncluttered clear and calming app interfaces that are not overstimulating.

#### Patient App Usability in Cycle 1

The mean score of the SUS for the patient app in cycle 1 was 81.7 (SD 20.1) out of a possible 100. Qualitative feedback on the patient app mostly included positive comments concerning the overall functionality and user interface design of the app. Almost all (8/9, 89%) the participants appreciated the color, design, and layout of the app. The colors were deemed as “calm” and “soothing” by the participants. The only significant concern was centered on the *help* button in the app. Several (3/9, 33%) participants initially thought that the help button was for technical support, rather than for crisis emergency contacts. In addition, participants had recommendations about the content for the Distractions page, such as adding puzzles, games, music, and relaxation videos. On the basis of participant recommendations, we updated the *Distractions* page by adding the aforementioned puzzles and relaxation videos. We also made some updates to the app, such as fixing typographical errors throughout, adding the app version to the menu section, and adding additional information about the program to the psychoeducation section.

### Cycle 2

#### Participant Characteristics in Cycle 2

A total of 11 patients were approached by the research assistant during the second cycle of usability testing. Of these 11 patients, 4 (36%) did not have an Android smartphone; 1 (27%) patient declined; 1 (27%) patient did not have a stable mailing address; and finally, 5 (45%) patients were enrolled. In cycle 2, 40% (2/5) of participants were women; 20% (1/5) of patients was a man, and 40% (2/5) of patients were nonbinary. In addition, 80% (4/5) of patients were identified as White and 20% (1/5) of patients as belonging to more than one race. The other 40% (2/5) of participants were Hispanic or Latino. The mean age of the participants was 26.4 (SD 8.4) years. In total, 80% (4/5) of participants reported being diagnosed with a psychiatric or emotional disorder. All 5 (100%) patients endorsed having thoughts of harming or killing themselves, and 80% (4/5) had attempted to kill or harm themselves at some point in their lives. All participants owned a smartphone and had brought their cell phones to the ED. Similar to cycle 1, none of the participants had access to their cell phones at the time of the research interview. All the participants endorsed using their cell phone for more than 3 hours each day. In terms of smartphone self-efficacy, all the participants agreed or strongly agreed that they could use smartphone technology if there was no one around to tell them what to do; 80% (4/5) of participants agreed or strongly agreed that they could use smartphone technology even if they had never used a similar technology before; and all 5 (100%) participants strongly agreed that they were confident that they could effectively open and use an app on a smartphone. These data show that the participants in this cycle were also represented by personas that were developed in iteration 1.

#### ED App Usability in Cycle 2

Similar to cycle 1, of the total 5 participants, 3 (60%) participants chose to view the clinician intro video; 1 (20%) chose the animated video; and 1 (20%) chose the community member video. Qualitative feedback on the ED app during cycle 2 was minimal; 1 (20%) participant still endorsed that asking individuals to list someone as social support could prove distressing to those without such ties. Otherwise, participants had no suggestions for further improvement.

All (5/5, 100%) the participants were able to initiate the videos and navigated successfully through the safety planning process. The mean score of the SUS in cycle 2 for the ED app increased to 90.5 (SD 9.9) out of a possible 100, indicating that the usability of the ED app was very high [30], and the UES score increased to 4.07 (SD 0.36) out of a possible 5. Analysis of UES subscales again showed that perceived usability (mean 5.00, SD 0.00) and reward factor (mean 4.20, SD 0.75) were very high and aesthetic appeal also improved (mean 4.33, SD 0.42), but focused attention remained low (mean 2.73, SD 0.68), as would be expected for this type of product and setting.

#### Patient App Usability in Cycle 2

The mean score of the SUS for the smartphone app in cycle 2 was 97.0 (SD 1.87) out of a possible 100. Similar to the ED app, qualitative feedback on the patient app collected during the interviews conducted at the ED in Cycle 2 was minimal. Beside commentary on esthetic choices, such as adding an additional color to the palette, participants made suggestions for improvements they would like to see in the future, such as ability to share access to their My Plans section with their regular therapist, a larger video selection for distractions including talk shows and art videos, and the ability to create a journal.

When cycle 2 patients were followed up after discharge, only 40% (2/5) of the participants reported downloading the app. Of the 3 who had not downloaded the app, 2 (67%) said that they had forgotten and 1 (33%) said that they had not seen the email containing the download link. Of the 2 participants who downloaded the app, both (100%) rated their downloading experience as 10 out of 10, indicating the best possible experience. Of 2 participants, 1 (50%) reported using the app once, and the other (50%) participant reported using it “a couple of times.” Both the participants endorsed using the safety plan and distractions the most. Of 2 patients, only 1 (50%) patient completed the structured measures at follow-up, with a SUS score of 95/100.

### Completeness of Safety Plans Created

During the safety planning process, 11 participants chose to provide meaningful content (as opposed to filler) to create a “real” safety plan in the ED app. We rated these 11 safety plans using the Brief Safety Plan Scoring Form. Each line of safety plan content was coded as absent (0 points), present but poor specificity (1 point), or present and sufficient (2 points); and the points summed to give an overall quality score for each safety plan—the mean score for safety plan quality was 27.4 (SD 3.4) out a possible 36—suggesting good overall quality of safety plans. Table 1 shows the completeness of these 11 safety plans by safety plan step, as well as the proportion of participants who added extra content to each step. The most incomplete step was step 5 (Professionals for a Crisis).

**Table 1:** Completeness of safety plans created in the ReachCare emergency department app (n=11).

Steps	Lines completed, mean/maximum possible (completeness rate, %)	Safety plans with additional content, n (%)
Step 1: Warning signs (3 lines)	2.8/3 (93)	4 (36)
Step 2: Coping strategies (3 lines)	2.9/3 (97)	4 (36)
Step 3: People and places for distraction (4 lines)	3.6/4 (90)	2 (18)
Step 4: Social contacts for a crisis (3 lines)	2.2/3 (73)	1 (9)
Step 5: Professionals for a crisis (3 lines)	1.3/3 (43)	1 (9)
Step 6: Making the environment safe (2 lines)	1.7/2 (85)	0 (0)

## Discussion

### Principal Findings

In this development and usability study, we created personas that represented the needs and challenges of our patient population. We used these personas to prioritize and inform our design decisions and draft initial designs. Our design goals for creating a successful patient experience included satisfactory subjective and objective usability measures. On the basis of the feedback from individuals with lived experience, we then created prototypes that received good usability and acceptability ratings from ED patients. The objective usability measure of task success also yielded satisfactory results: all ED patients were able to initiate viewing the provided videos and use the chatbot to navigate through the safety planning process. These findings suggest that our iterative user-centered approach was effective in developing a satisfactory experience for patients using ReachCare apps.

We leveraged personas to design for common needs rather than for a specific user group. The personas suggested that we ought to minimize cognitive effort and facilitate a calm, caring, supportive, and welcoming experience. We also used these personas to draft the initial designs that we used to solicit additional information about patient preferences and their practical and emotional needs. There is growing evidence that personas offer benefits over alternative approaches (such as analytics systems) in terms of efficiency, learnability, and consistency [31] and may be especially helpful in designing health-related interventions [32]. Our approach to UCD may be especially helpful for suicide-related interventions, given the unique and dynamic states of mind involved in suicidal crises. However, few studies have applied UCD for suicide interventions; many published studies of UCD in mental health to date have focused on lower acuity concerns such as depression and anxiety [33]. Our goal was to center on lived experience [34] and patient autonomy in intervention development, for example, by offering choices where possible without overwhelming the patient in crisis. The prototypes demonstrated good usability and acceptability among ED patients with suicide risk, with ratings improving across the iterative cycles. According to the user-centered approach to technology design, users’ subjective experience of digital applications plays a significant role in deciding whether to adopt or continue using a technology [16]. Hence, we relied primarily on subjective user evaluations to benchmark design improvement in our study. We chose the SUS as a subjective measure of usability in our study because of its suitability for benchmarking digital health applications [35]. In terms of task completion, the safety plans generated by the patients were, in large part, of good quality and completeness, apart from an apparent dearth of professionals that participants could turn to in a crisis. This finding, similar to that of earlier research, supports the feasibility of self-administered safety planning in the ED [14]. ReachCare provides supportive design and additional functionality, including helplines, distractions, referral resources, and self-care education. Our study revealed some interesting patterns in subjective user engagement, where we found high scores for the reward factor, perceived usability, and esthetic appeal but low scores for focused attention. Possible explanations are the crisis nature of ED visits, which naturally reduces one’s ability to focus or that the items used for capturing focused attention are not calibrated adequately for this context. Focused attention items in the UES are aligned with the concept of *flow* (eg, I lost my self or was absorbed in the experience, time slipped away) which refers to a universally experienced state of enjoyment [36]. This is an emotional state that patients who are at risk for suicide are unlikely to experience, particularly during an ED visit. Indeed, our qualitative data also showed that focused attention is a challenge for our target users. This highlights the importance of qualitative data collection during usability assessment iterations, where the subconstructs address sensitive issues that are not commonly observed in typical consumer-facing digital technologies. Although these scores represent the summarization of nuanced and complex experiences observed in our context, the addition of qualitative data is crucial for translating these scores to design decisions that lead to improvement. Overall, we recommend that usability and engagement scales be coupled with qualitative interviews to better interpret scores and make effective design decisions, especially for users with complex mental health needs.

### Limitations

In terms of limitations of this study, the sample size was small and the design was mostly cross sectional. Moreover, we did not have an opportunity to engage family members during the index ED encounters. A minority of those approached in the ED participated in the usability study, and our sample skewed toward younger population than expected, suggesting that mobile apps may be less attractive, acceptable, or applicable to older ED patients. Finally, we relied primarily on self-report scales and task completion rather than other task metrics (eg, task completion time) for our quantitative assessment of usability. Recent research has suggested that task completion may have stronger correlations with usability benchmarks than the SUS does [37]. Hence, using both SUS and task completion in usability studies provides complementary feedback on important design elements. Despite these shortcomings, we did apply a user-centered approach, engaged a range of stakeholders in development, and tested usability under naturalistic conditions.

### Conclusions

Future studies, including those carried out by our research team, can extend this work by designing personalized experiences that address the unique needs of different user groups and opportunities for enhancement to improve engagement [38]. ReachCare builds on our team’s previous research, including a trial that established the clinical effectiveness of the ED-SAFE intervention [7], a computer-administered safety planning application [14], and an implementation study of the safety planning intervention [24]. Our results support the feasibility and acceptability of technology-assisted delivery of interventions for ED patients who are at risk for suicide, such as safety planning and caring contacts. We anticipate that ReachCare will address some of the trenchant barriers to implementing evidence-based care for suicidality during and after an ED presentation.

## Abbreviations

ED: emergency department
ED-SAFE: Emergency Department Safety Assessment and Follow-up Evaluation
SUS: System Usability Scale
UCD: user-centered design
UES: User Engagement Scale

## Data Availability

Data will be available in the National Institute of Mental Health Data Archive after completion of the parent study.
